# Supplementary material for: A lipid cue drives the subcellular localization of a self-inserting bacterial transmembrane protein
Source: bioRxiv. 2026 May 29:2026.05.27.728190. Preprint. [Version 1] doi: 10.64898/2026.05.27.728190 (PMC13232292; doi:10.64898/2026.05.27.728190)
Supplement: Supplement 1 [file NIHPP2026.05.27.728190v1-supplement-1.pdf]

bioRxiv preprint doi: https://doi.org/10.64898/2026.05.27.728190; this version posted May 29, 2026. The copyright holder for this preprint (which was not certified by peer review) is the author/funder. This article is a US Government work. It is not subject to copyright under 17 USC 105 and is also made available for use under a CC0 license.

Supporting Information for

A lipid cue drives the subcellular localization of a self-inserting bacterial transmembrane protein

Vani Pande, Taylor B. Updegrove, Vivek Anantharaman, Ashley Bae, Jiji Chen, L. Aravind, and Kumaran S. Ramamurthi*

*Corresponding author: Kumaran S. Ramamurthi
Email: ramamurthiks@mail.nih.gov

This PDF file includes:

Figure S1-S3
Table S1

bioRxiv preprint doi: https://doi.org/10.64898/2026.05.27.728190; this version posted May 29, 2026. The copyright holder for this preprint (which was not certified by peer review) is the author/funder. This article is a US Government work. It is not subject to copyright under 17 USC 105 and is also made available for use under a CC0 license.

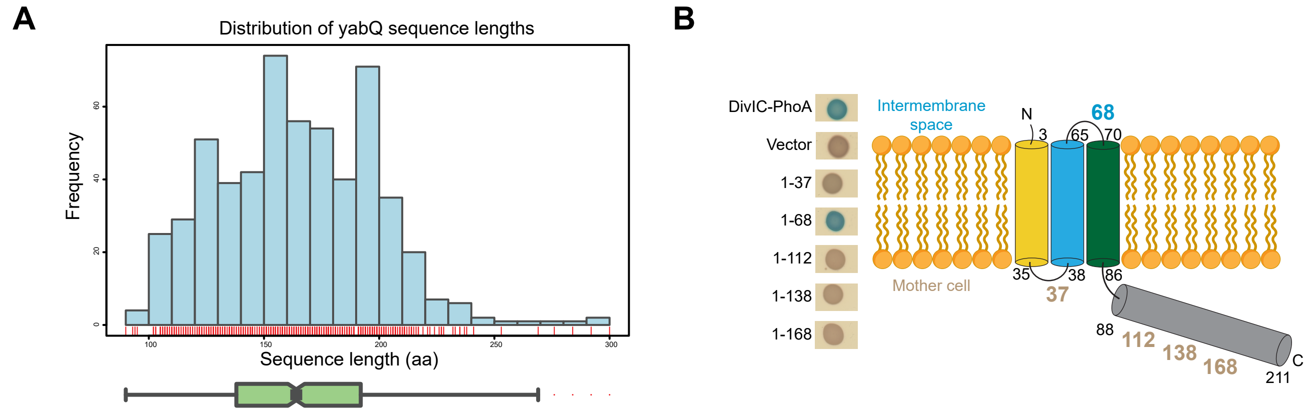

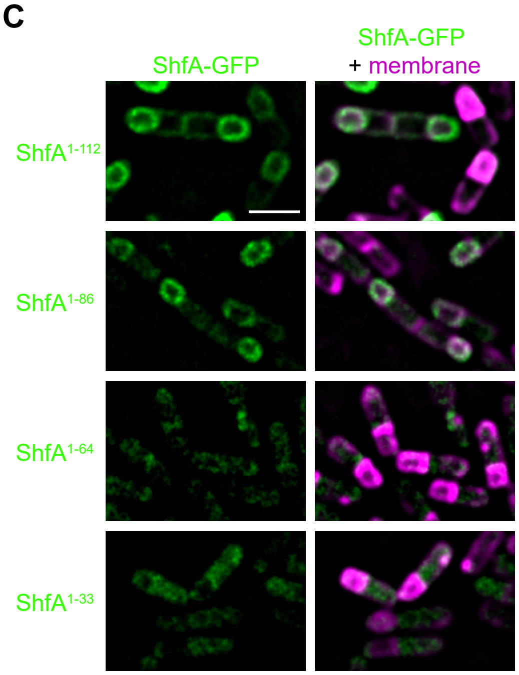

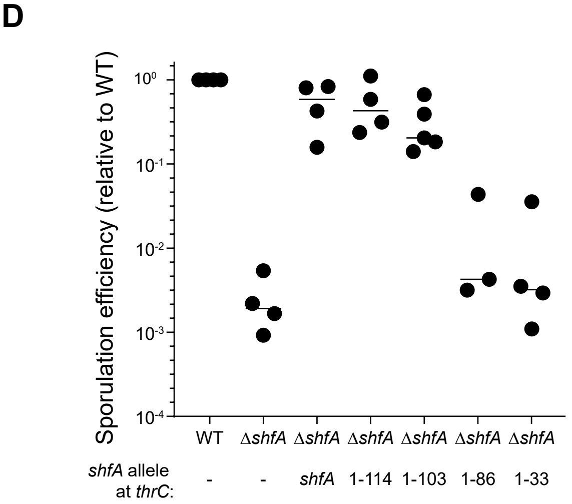

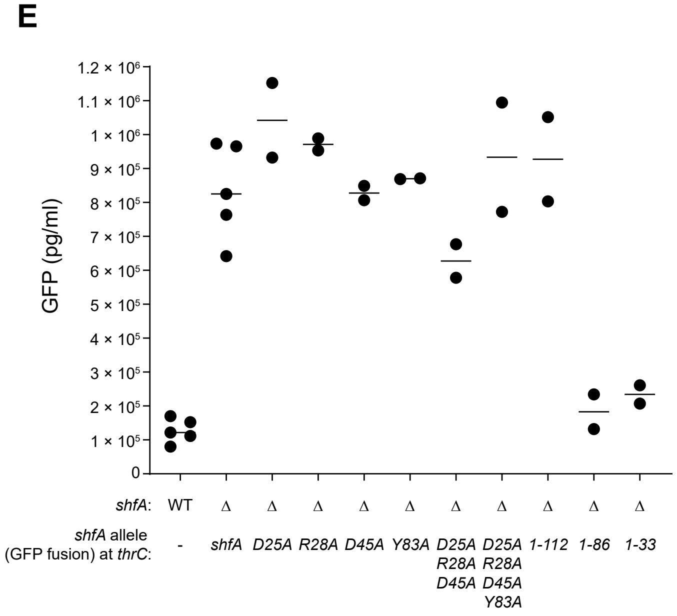

Figure S1. The YabQ domain of ShfA is important for function and subcellular localization. (A) Histogram of ShfA protein lengths in amino acids from a curated, nonredundant set of complete genomes illustrates variability across species. A rug plot along the X-axis (red) indicates the density of observations in each bin, and a green box plot below shows the length distribution with red dots marking outliers. (B) Left: representative *E. coli* colonies harboring indicated fusions of ShfA to PhoA and plated on agar containing 5-bromo-4-chloro-3-indolyl phosphate to indicate PhoA activity (blue color indicates high PhoA activity). Right: Schematic of the inferred ShfA membrane topology. Amino acid positions are indicated; fusions to ShfA residues indicated in brown resulted in low PhoA activity, suggesting cytosolic localization of PhoA; fusion to ShfA residue indicated in blue resulted in high PhoA activity, suggesting extracytosolic localization of PhoA. C-terminal fusion of PhoA to DivIC is a positive control for extracytosolic localization of PhoA; "Vector" is a negative control for cytosolic localization of PhoA. Strains: VP254, VP273, VP274, VP275, VP276, VP277 and VP278.(C) Subcellular localization of indicated ShfA-GFP truncations 3.5 h after induction of sporulation. Left: fluorescence from ShfA-GFP; right: overlay, GFP and membranes. Strains: CW214, CW212, CW213, CW211. (D) Sporulation efficiencies relative to WT for strains harboring the indicated truncation allele of *shfA*. *thrC* is a chromosomal locus in which different alleles of *shfA* are inserted. Bars represent mean; data points indicate independent experiments. (E) Intracellular accumulation of indicated ShfA-GFP variant produced from the *thrC* locus at 3.5 after induction of sporulation as determined by ELISA.

bioRxiv preprint doi: https://doi.org/10.64898/2026.05.27.728190; this version posted May 29, 2026. The copyright holder for this preprint (which was not certified by peer review) is the author/funder. This article is a US Government work. It is not subject to copyright under 17 USC 105 and is also made available for use under a CC0 license.

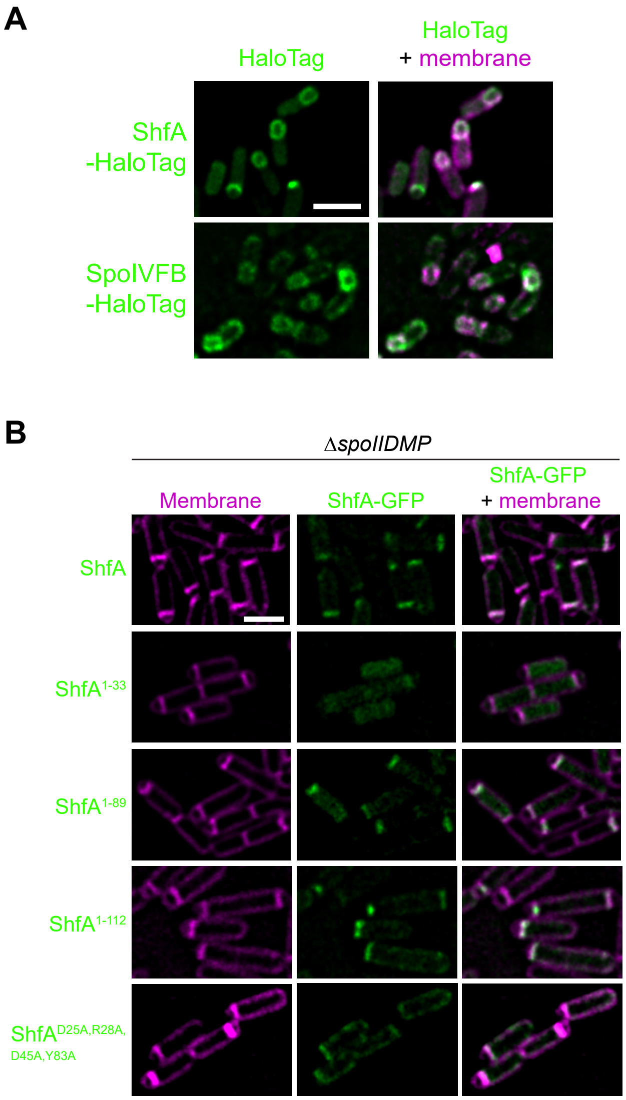

Figure S2. ShfA-GFP can localize to a flat asymmetric septum during sporulation. (A) Subcellular localization of ShfA-HaloTag and SpoIVFB-HaloTag in sporulating *B. subtilis* strains 3.5 h after induction of sporulation in the presence of excess JFX554 dye (100 nM). Left: fluorescence from JFX554 dye bound HaloTag; right: overlay, HaloTag and membranes visualized with MitoTracker Deep Red FM. Strains: VP461 and VP462. Scale bar: 2 μm. (B) Subcellular localization of indicated ShfA-GFP truncations or variant 3.5 h after induction of sporulation in a Δ*spoIID/M/P* strain. Left: membranes visualized using FM4-64; center: fluorescence from ShfA-GFP; right: overlay, GFP and membranes. Scale bar: 2 μm.

bioRxiv preprint doi: https://doi.org/10.64898/2026.05.27.728190; this version posted May 29, 2026. The copyright holder for this preprint (which was not certified by peer review) is the author/funder. This article is a US Government work. It is not subject to copyright under 17 USC 105 and is also made available for use under a CC0 license.

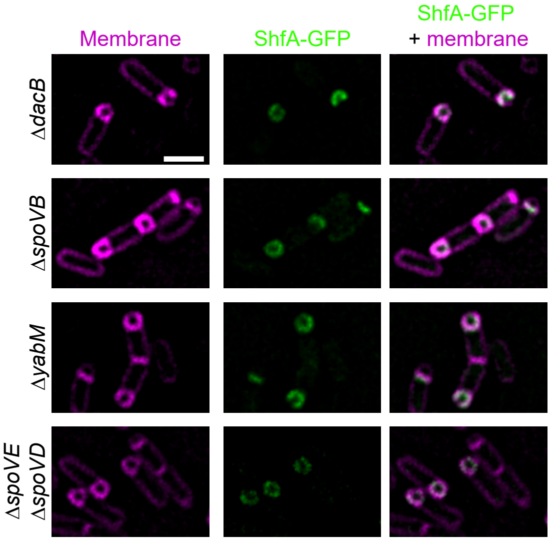

Figure S3. Spore cortex peptidoglycan remodeling proteins do not determine ShfA localization. Subcellular localization of ShfA-GFP 2.5 h after induction of sporulation in the absence of *dacB*, *spoVB*, *yabM*, or in a Δ*spoVE* Δ*spoVD* double mutant. Left: membranes visualized using FM4-64; center: fluorescence from ShfA-GFP; right: overlay, GFP and membranes. Strains: VP118, VP131, VP123, VP137. Scale bar: 2 µm.

bioRxiv preprint doi: https://doi.org/10.64898/2026.05.27.728190; this version posted May 29, 2026. The copyright holder for this preprint (which was not certified by peer review) is the author/funder. This article is a US Government work. It is not subject to copyright under 17 USC 105 and is also made available for use under a CC0 license.

Table S1. Bacterial strains used in this study

Bacillus subtilis

Strain	Genotype or description	Reference
PY79	Prototrophic derivative of *B. subtilis* 168	(1)
CW202	*ΔyabQ::tet*	(2)
VP088	*ΔyabQ::tet thrC::*P_{yab}*-yabQ spec*	This study
CW201	*thrC::*P_{yab}*-yabQ-gfp spec*	This study
CW205	*ΔyabQ::tet thrC::*P_{yab}*-yabQ-gfp spec*	This study
CVO1251	*ΔspoIIAC::erm amyE::*P_{spoIIE}*-spoIIR neo*	(3)
VP010	*ΔspoIIAC::erm amyE::*P_{spoIIE}*-spoIIR neo thrc::*P_{yab}*-yabQ-gfp spec*	This study
CW189	*amyE::*$P_{hyperspank}$*-yabQ-gfp spec*	This study
VP050	*ΔsinI::kan amyE::*$P_{hyperspank}$*-yabQ-gfp spec*	This study
BDR647	*amyE::*P_{xylose}*-spoIVFB-gfp cat*	(4)
VP093	*ΔdacA::kan*	(5)
CC248	*ΔyabQ::erm*	This study
DPVB488	*ΔyabM::erm*	(6)
KR585	*ΔspoVB::tet*	(7)
KR582	*ΔspoVD::erm*	This study
KR587	*ΔspoVE::kan*	(7)
FC332	*ΔsinI::kan*	(8)
PE234	*ΔdacB::tet*	(7)
KP73	*ΔspoIVA::neo*	(9)
VP096	*ΔdacA::kan amyE::*$P_{hyperspank}$*-yabQ-gfp spec*	This study
VP118	*ΔdacB::tet ΔyabQ::erm thrC::*P_{yab}*-yabQ-gfp spec*	This study
VP123	*ΔyabQ::tet ΔyabM::erm thrC::*P_{yab}*-yabQ-gfp spec*	This study
VP131	*ΔspoVB::tet ΔyabQ::erm thrC::*P_{yab}*-yabQ-gfp spec*	This study
VP137	*ΔyabQ::tet ΔspoVD::erm ΔspoVE::kan thrC::*P_{yab}*-yabQ-gfp spec*	This study
VP375	*ΔspoIVA::neo thrC::* P_{yab} *-yabQ-gfp spec*	This study
CC10	*ΔyabQ::tet thrC::*P_{yab} *-yabQ*^{D25A}*-gfp spec*	This study
CC11	*ΔyabQ::tet thrC::*P_{yab} *-yabQ*^{R28A}*-gfp spec*	This study
CC12	*ΔyabQ::tet thrC::*P_{yab} *-yabQ*^{D45A}*-gfp spec*	This study
CC13	*ΔyabQ::tet thrC::*P_{yab}*-yabQ*^{Y83A}*-gfp spec*	This study
CC30	*ΔyabQ::tet thrC::*P_{yab}*-yabQ*^{D25A,R28A,Y83A}*-gfp spec*	This study
VP069	*ΔyabQ::tet thrC::*P_{yab}*-yabQ*^{D25A,R28A,D45A,Y83A}*-gfp spec*	This study
VP298	*ΔyidC1::kan ΔyidC2::erm ΔyabQ::tet thrC::*P_{yab}*-yabQ-gfp spec*	This study
NKB150	*divICΩspec*	(10)
VP350	*amyE::*$P_{hyperspank}$*-yabQ-gfp spec::cat yabQ::erm divICΩspec*	This study
VP428	*amyE::* $P_{hyperspank}$*-RBS-MreB spec*	This study
VP433	*amyE::* $P_{hyperspank}$*-mreB spec thrC::*$P_{hyperspank}$*-yabQ-gfp erm*	This study
BIR634	*ΔsacA::*P_{veg}*-mTagBFP phleo ΔamyE::*P_{amj}*-yfp cat ΔuppS::tet ΔycgO::*$P_{hyperspank}$*-uppS spec*	(11)
VP409	*thrC::*P_{yab}*-yabQ-gfp spec::erm*	This study

bioRxiv preprint doi: https://doi.org/10.64898/2026.05.27.728190; this version posted May 29, 2026. The copyright holder for this preprint (which was not certified by peer review) is the author/funder. This article is a US Government work. It is not subject to copyright under 17 USC 105 and is also made available for use under a CC0 license.

VP418	*thrC::P_{yab}-yabQ-gfp spec::erm ycgO::$P_{hyperspank}$-uppS spec*	This study
VP422	*thrC::P_{yab}-yabQ-gfp spec::erm ycgO::$P_{hyperspank}$-uppS spec uppS::tet*	This study
CW214	*ΔyabQ::tet thrC::P_{yab}-yabQ$^{1\text{-}33}$-gfp spec*	This study
CW212	*ΔyabQ::tet thrC::P_{yab}-yabQ$^{1\text{-}86}$-gfp spec*	This study
CW213	*ΔyabQ::tet thrC::P_{yab}-yabQ$^{1\text{-}64}$-gfp spec*	This study
CW211	*ΔyabQ::tet thrC::P_{yab}-yabQ$^{1\text{-}112}$-gfp spec*	This study
VP088	*ΔyabQ::tet thrC::P_{yab}-yabQ spec*	This study
VP182	*ΔyabQ::tet thrC::P_{yab}-yabQ^{D25A} spec*	This study
VP183	*ΔyabQ::tet thrC::P_{yab}-yabQ^{D45A} spec*	This study
VP185	*ΔyabQ::tet thrC::P_{yab}-yabQ^{R28A} spec*	This study
VP189	*ΔyabQ::tet thrC::P_{yab} -yabQ^{Y83A} spec*	This study
VP197	*ΔyabQ::tet thrC::P_{yab}-yabQ^{D25A,R28A,D45A} spec*	This study
VP210	*ΔyabQ::tet thrC::P_{yab}-yabQ^{D25A,R28A,D45A,Y83A} spec*	This study
VP192	*ΔyabQ::tet thrC::P_{yab}-yabQ$^{1\text{-}114}$spec*	This study
VP193	*ΔyabQ::tet thrC::P_{yab}-yabQ$^{1\text{-}103}$spec*	This study
VP194	*ΔyabQ::tet thrC::P_{yab}-yabQ$^{1\text{-}86}$spec*	This study
VP196	*ΔyabQ::tet thrC::P_{yab}-yabQ$^{1\text{-}33}$spec*	This study
VP075	*ΔspoIID::cat ΔspoIIP::kan ΔspoIIM::Tn917ΩHU287::erm ΔyabQ::tet thrC::P_{yab}-yabQ-gfp spec*	This study
VP234	*ΔspoIID::cat, ΔspoIIP::kan, ΔspoIIM::Tn917ΩHU287::erm ΔyabQ::tet thrC::P_{yab}-yabQ$^{1\text{-}112}$-gfp spec*	This study
VP235	*ΔspoIID::cat, ΔspoIIP::kan, ΔspoIIM::Tn917ΩHU287::erm ΔyabQ::tet thrC::P_{yab}-yabQ$^{1\text{-}86}$-gfp spec*	This study
VP236	*ΔspoIID::cat, ΔspoIIP::kan, ΔspoIIM::Tn917ΩHU287::erm ΔyabQ::tet thrC::P_{yab}-yabQ$^{1\text{-}33}$-gfp spec*	This study
VP237	*ΔspoIID::cat, ΔspoIIP::kan, ΔspoIIM::Tn917ΩHU287::erm ΔyabQ::tet thrC::P_{yab}-yabQ^{D25A,R28A,D45A,Y83A}-gfp spec*	This study
VP461	*amyE:: $P_{hyperspank}$ -shfA-HaloTag spec*	This work
VP462	*amyE:: $P_{hyperspank}$ --spoIVFB-HaloTag spec*	This work

Staphylococcus aureus

Strain	Genotype or description	References
RN4220	*pVP012 (pJB67* backbone, *P_{CdCl2}-yabQ-gfp)*	This study

***Escherichia coli* strains used in this study**

Strain	Genotype or description	References
BL21(DE3)	*pVP008 (pET28a* backbone, *P_{T7}-yabQ-gfp-his6)*	This study
VP254	*pKTop* empty vector	(12)
VP273	*pVP038 (pKTop backbone, yabQ $^{1\text{-}37}$)*	This study
VP274	*pVP039 (pKTop backbone, yabQ $^{1\text{-}68}$)*	This study
VP275	*pVP040 (pKTop backbone, yabQ $^{1\text{-}112}$)*	This study
VP276	*pVP041 (pKTop backbone, yabQ $^{1\text{-}138}$)*	This study
VP277	*pVP042 (pKTop backbone, yabQ $^{1\text{-}168}$)*	This study

bioRxiv preprint doi: https://doi.org/10.64898/2026.05.27.728190; this version posted May 29, 2026. The copyright holder for this preprint (which was not certified by peer review) is the author/funder. This article is a US Government work. It is not subject to copyright under 17 USC 105 and is also made available for use under a CC0 license.

VP278	*pVP043 (pKTop* backbone, *divIC$^{full\ length}$)*	This study

REFERENCES

1. P. Youngman, J. B. Perkins, R. Losick, Construction of a cloning site near one end of Tn917 into which foreign DNA may be inserted without affecting transposition in *Bacillus subtilis* or expression of the transposon-borne erm gene. *Plasmid* **12**, 1–9 (1984).
2. T. B. Updegrove *et al.*, Altruistic feeding and cell-cell signaling during bacterial differentiation actively enhance phenotypic heterogeneity. *Sci Adv* **10**, eadq0791 (2024).
3. C. van Ooij, P. Eichenberger, R. Losick, Dynamic patterns of subcellular protein localization during spore coat morphogenesis in *Bacillus subtilis*. *J Bacteriol* **186**, 4441–4448 (2004).
4. D. Z. Rudner, Q. Pan, R. M. Losick, Evidence that subcellular localization of a bacterial membrane protein is achieved by diffusion and capture. *Proc Natl Acad Sci U S A* **99**, 8701–8706 (2002).
5. S. Sanchez, E. V. Snider, X. Wang, D. B. Kearns, Identification of Genes Required for Swarming Motility in *Bacillus subtilis* Using Transposon Mutagenesis and High-Throughput Sequencing (TnSeq). *J Bacteriol* **204**, e0008922 (2022).
6. P. Vasudevan, J. McElligott, C. Attkisson, M. Betteken, D. L. Popham, Homologues of the *Bacillus subtilis* SpoVB protein are involved in cell wall metabolism. *J Bacteriol* **191**, 6012–6019 (2009).
7. P. Eichenberger, P. Fawcett, R. Losick, A three-protein inhibitor of polar septation during sporulation in *Bacillus subtilis*. *Mol Microbiol* **42**, 1147–1162 (2001).
8. D. B. Kearns, F. Chu, S. S. Branda, R. Kolter, R. Losick, A master regulator for biofilm formation by *Bacillus subtilis*. *Mol Microbiol* **55**, 739–749 (2005).
9. S. Roels, A. Driks, R. Losick, Characterization of *spoIVA*, a sporulation gene involved in coat morphogenesis in *Bacillus subtilis*. *J Bacteriol* **174**, 575–585 (1992).
10. N. King, O. Dreesen, P. Stragier, K. Pogliano, R. Losick, Septation, dephosphorylation, and the activation of sigmaF during sporulation in *Bacillus subtilis*. *Genes Dev* **13**, 1156–1167 (1999).
11. I. J. Roney, D. Z. Rudner, *Bacillus subtilis* uses the SigM signaling pathway to prioritize the use of its lipid carrier for cell wall synthesis. *PLoS Biol* **22**, e3002589 (2024).
12. G. Karimova, D. Ladant, Defining Membrane Protein Topology Using pho-lac Reporter Fusions. *Methods Mol Biol* **2715**, 181–195 (2024).
